# Different involvement of medial prefrontal cortex and dorso-lateral striatum in automatic and controlled processing of a future conditioned stimulus

**DOI:** 10.1371/journal.pone.0189630

**Published:** 2017-12-14

**Authors:** Francisco Pérez-Díaz, Estrella Díaz, Natividad Sánchez, Juan Pedro Vargas, John M. Pearce, Juan Carlos López

**Affiliations:** 1 Animal Behav & Neurosci Lab, Dpt. Psicología Experimental, Universidad de Sevilla, c/ Camilo Jose Cela s/n, Seville, Spain; 2 School of Psychology, Cardiff University, Cardiff, Wales, United Kingdom; 3 School of Psychology, University of Sydney, Sydney, Australia; Technion Israel Institute of Technology, ISRAEL

## Abstract

Recent studies support the idea that stimulus processing in latent inhibition can vary during the course of preexposure. Controlled attentional mechanisms are said to be important in the early stages of preexposure, while in later stages animals adopt automatic processing of the stimulus to be used for conditioning. Given this distinction, it is possible that both types of processing are governed by different neural systems, affecting differentially the retrieval of information about the stimulus. In the present study we tested if a lesion to the dorso-lateral striatum or to the medial prefrontal cortex has a selective effect on exposure to the future conditioned stimulus (CS). With this aim, animals received different amounts of exposure to the future CS. The results showed that a lesion to the medial prefrontal cortex enhanced latent inhibition in animals receiving limited preexposure to the CS, but had no effect in animals receiving extended preexposure to the CS. The lesion of the dorso-lateral striatum produced a decrease in latent inhibition, but only in animals with an extended exposure to the future conditioned stimulus. These results suggest that the dorsal striatum and medial prefrontal cortex play essential roles in controlled and automatic processes. Automatic attentional processes appear to be impaired by a lesion to the dorso-lateral striatum and facilitated by a lesion to the prefrontal cortex.

## Introduction

Latent inhibition (LI) is a retardation in the acquisition of a conditioned response during Pavlovian conditioning, if the CS has previously been presented on its own [1]. A variety of theoretical explanations have been offered for this effect. Some of these theories attribute LI to retrieval failure [2–4], other theories attribute it to acquisition failure [5–8]. With regard to the latter, it has been proposed on more than one occasion that the acquisition failure results from a loss of attention to the CS [9, 10]. For example, according to Pearce and Hall [7] when the significance of a stimulus is uncertain, such as when it is novel, or during the early stages of conditioning, then it will receive controlled processing that will enable leaning about the stimulus. However, when the significance of a stimulus is certain, such as when it has been repeatedly followed by nothing or by the same US, then it will receive automatic processing [7], which will make it difficult for further learning about the stimulus to take place [11–13]. Even though both process are well defined, none of the current associative model predict when processing will became automatic during preexposure learning. Thus, once it is accepted that automatic and controlled attentional processes influence the course of learning, it is then important to identify the role played by different regions of the brain in these processes. The purpose of the present article is to explore the role played by the dorsal striatum and the medial prefrontal cortex.

In recent years, several studies have aided in the growth in our understanding of the role for striatal-frontal interactions supporting higher cognitive functions like attention or cognitive control of flexible actions [14–26]. Corticostriatal loops have been proposed to be involved in these functions, even though studies focused specifically on attention have been scarce. Recently, we argued that the dorsal striatum supports the cognitive control of memory retrieval. Díaz et al. [10] analyzed the role of dorsal striatum in retrieval processes of LI using different amounts of exposure to the CS. More specifically, one group of rats was exposed to many presentations of the future CS of saccharine without consequences, over a period of five days, while a second group received only two days of preexposure treatment. Both groups then received a single trial of taste aversion conditioning with saccharine, before being tested with saccharine by itself. Critically, the dorso-lateral striatum (dls) was blocked during the final test phase. The results showed that the blockade of dls during the test phase disrupted LI in the group with five days of preexposure, but not in the group with two days of preexposure. It was concluded that successful LI, after extensive preexposure, depends upon automatic processing of the relevant stimulus, and that this processing is effective after many, but not a few preexposure trials. If this conclusion is correct, then a similar outcome will be observed if the dls is rendered inactive before the preexposure training, rather than before the test trial. One purpose of the present experiments is to test this prediction. An implication of the study by Diaz et al. [10], is that a mechanism other than automatic processing is responsible for LI after a few trials. One possibility is that some aspect of controlled processing, which is presumably directed to a stimulus after a few exposure trials, is responsible for any loss in associability that might be observed. A further purpose of the experiments is to explore whether the medial prefrontal cortex (mpfc) plays a role in this type of processing.

The mpfc has previously been implicated in the attribution of incentive value to stimuli and goal directed behavior [27–29]. The mpfc sends a dense glutamatergic projection to nucleus accumbens, a structure that is itself necessary for LI expression. In addition, corticostriatal loops include projections from mpfc to dorso-medial striatum (dms), which work together in goal directed behavior [30, 31]. While these findings are compelling, the role of mpfc in controlled and automatic processes of a future CS has not yet been investigated.

Here, we analyzed the involvement of dls and mpfc in attentional processes. Specifically, we tested if dls and mpfc are involved in automatic and controlled processes by assessing dls and mpfc lesions in rats followed by extended and limited exposure to a future CS in the conditioned taste aversion paradigm (CTA). Specifically, we analyzed if lesion to these structures resulted in selective deficits that were dependent upon the duration of exposure to future CS. If dls is involved in the automatic stimulus processing, the subjects should display a decreased LI with extended exposure. In contrasts, lesion to this structure should not affect the performance after limited exposure, where the controlled attentional processes would facilitate a normal LI expression. On the other hand, if the mpfc is involved in controlled attentional processes, the lesion should facilitate automatic processes, and it should increase the LI expression in a limited exposure. Taken together, these studies will further the understanding of the role of the mpfc and dls in controlled and automatic processes of LI.

## General methods

### Subjects

174 adult male Wistar rats (300-400g) were used in these experiments, but 51 were excluded after histological analysis (see section below). All the animals were obtained from the Centro de Producción y Experimentación Animal (Universidad de Sevilla). Animals were individually housed in plastic cages (35×20×20 cm). The controlled temperature of the room was 21°C. The room was illuminated by four 100-W halogen lamps with a 14h light and 10h dark cycle. Rats were maintained and used for experimentation in accordance with the Guidelines of the European Union Council established by the Directive 2010/63/EU, and following the Spanish regulations (R.D 53/02013) for the use of laboratory animals. An ethical commission of University of Seville and Institutional Animal Care and Use Committee (Sanidad Animal, Junta de Andalucía) supervised and approved all the procedures and all protocols used in the this specific study. The code of the supervision report is: 31/08/2016/153. All animals were randomly distributed among the experimental conditions.

### Surgery

Under deep isoflurane anesthesia (2–5% in air, flow rate 1 l/min, 5% induction; 2% maintenance; McKinley type 2, Everest), rats were placed in a David Kopf stereotaxic instrument. The skin was incised to expose the skull, and following coordinates from Paxinos & Watson [32]: 1.6; 1.1; 0.6; and 0.1mm anterior to bregma; ±3.2; 3.5; 4.0 and 4.0mm lateral to the midline, and 5.0mm ventral to brain surface for dls lesion (for animals of experiment 1A and 1B), and 4.7; 4.2; 3.7mm anterior, ±0.6 lateral, and 4.0mm ventral for mpfc lesion (for animals of experiment 2A and 2B). The dls and mpfc lesion was made with NMDA (1 mg in 0.1 ml phosphate buffered saline, PBS 0.1M). The drug was injected into the brain through a 10μl Hamilton syringe (Model 1701 RN). The amount of NMDA solution injected at each site was 0.25 μl, at a rate of 0.05μl/min. The needle was left in place for an additional minute after the infusion to allow the diffusion of the solution into the tissue. No unexpected adverse effects exceeding moderate severity were observed 48 h after the surgery. Sham animals received a similar manipulation but no injection of the drug was carried out.

### Behavioral procedure

Behavioral procedures used were similar to that described in Díaz et al. [10]. After a one-week recovery period, the rats were randomly distributed among the experimental groups that received different amounts of exposure to the the future CS (see next paragraph). Three days before the preexposure phase, a deprivation program was implemented. Animals were allowed access to water for 10 min at a time at 10.00, 14.00 and 18.00 h for this period. All behavioral sessions were conducted in the vivarium. The water, and the saccharine (0.04%) that was used during the experiment was administered at room temperature in 150 ml glass bottles with stainless-steel mouthpieces. The bottles were attached to a frontal aperture in the grill of the home cage so that they remained immobile during the experimental session. The quantity of liquid consumed was calculated by weighing the bottle before and after each trial.

#### Extended exposure to the future CS

This phase lasted five days and was used in Experiments 1A and 2A. During this stage, all animals were allowed 10 min of access to water or saccharine 3 times per day (at 10.00 h, 14.00 h and 18.00 h). Rats were assigned to two different conditions. The extended exposure groups (E-exp) received saccharine three times a day for five days during this phase (dls lesion n = 7, dls sham n = 7; mpfc lesion n = 8, mpfc sham n = 8). The non-exposure groups to future CS (N-exp) were allowed 10 min access to water three times a day (dls lesion n = 7, dls sham n = 7; mpfc lesion n = 6, mpfc sham n = 6). After this period, the bottles were removed for all groups.

#### Limited exposure to the future CS

In experiment 1B and 2B, the groups given limited exposure group to the future CS (L-exp) received saccharine three times a day for two days during this phase (dls lesion n = 10, dls sham n = 10; mpfc lesion n = 13, mpfc sham n = 10). For the previous three days, they consumed only water. The groups given non-exposure to saccharine (N-exp) were just given water during the five days of this stage (dls lesion n = 6, sham n = 6; mpfc lesion n = 6, sham n = 6).

#### Conditioning

This phase consisted of a single session that took place on the day after the completion of the preexposure phase. All animals received only saccharine at 10.00 h for 10 min, followed by an intraperitoneal injection of LiCl dissolved in saline (0.4 M, 0.5% body weight). During the same day, at 14.00 and 18.00 hr, all groups were allowed 10 min access to water.

#### Test

This phase was run at 10.00 h on the day after conditioning. During this phase all rats had access to the saccharine solution for 10 min. Saccharine consumption for this trial reflected the level of taste aversion. As for conditioning, the treatment in this phase was the same in all the experiments.

### Histological analysis. Assessment of mpfc and dls lesion placement

**Fig 1:**
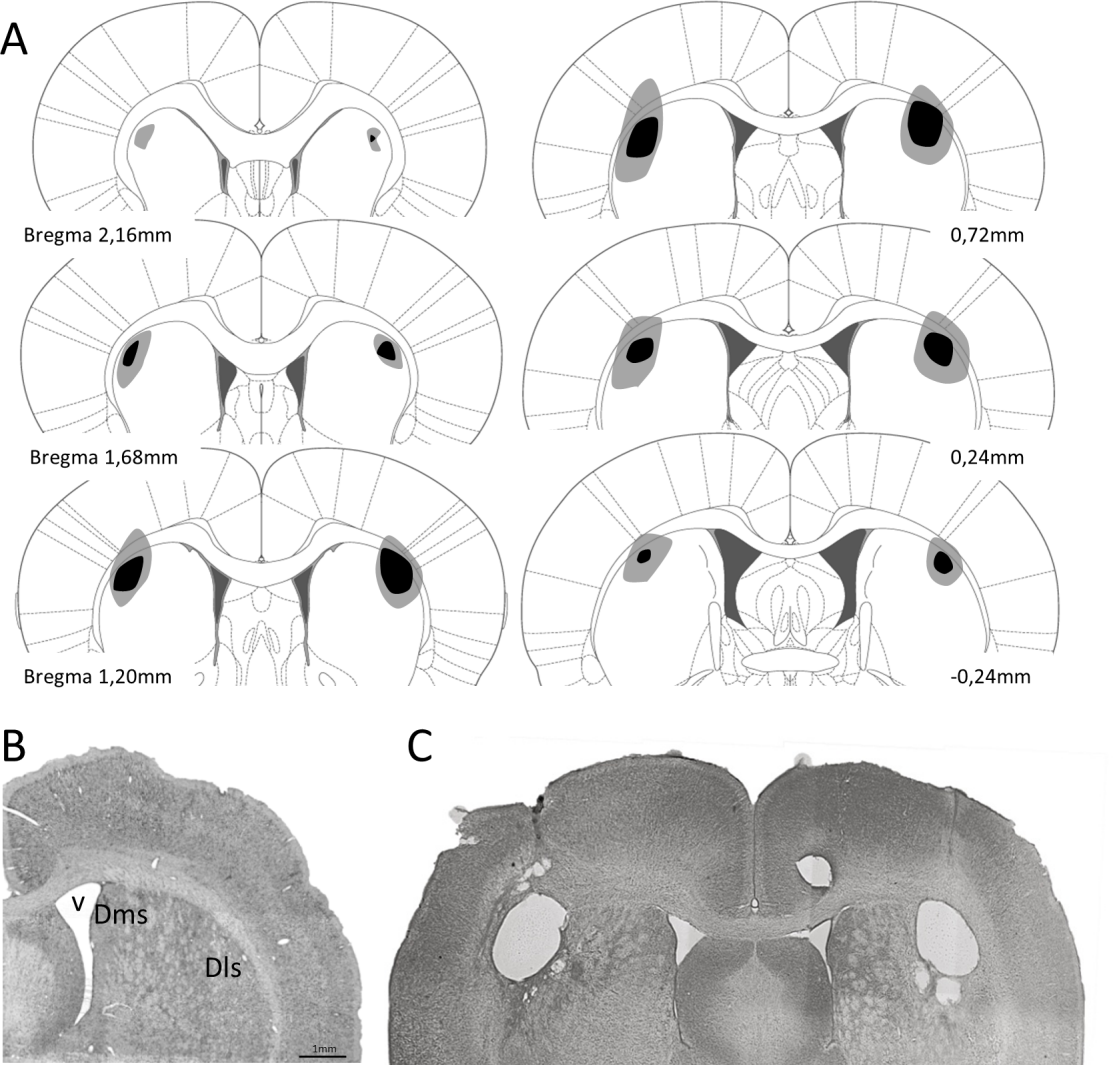
**A.** Reconstruction of the dls lesions displayed on standard coronal sections from the atlas of Paxinos and Watson [35]. The largest lesion is shown in pale shading and the smallest in dark shading. **B**. Photomicrograph showing a no lesioned coronal brain section and (**C**) a coronal section after excitotoxic lesion of dls. Dls: dorso-lateral striatum; Dms: dorso-medial striatum; v: lateral ventricle.

**Fig 2:**
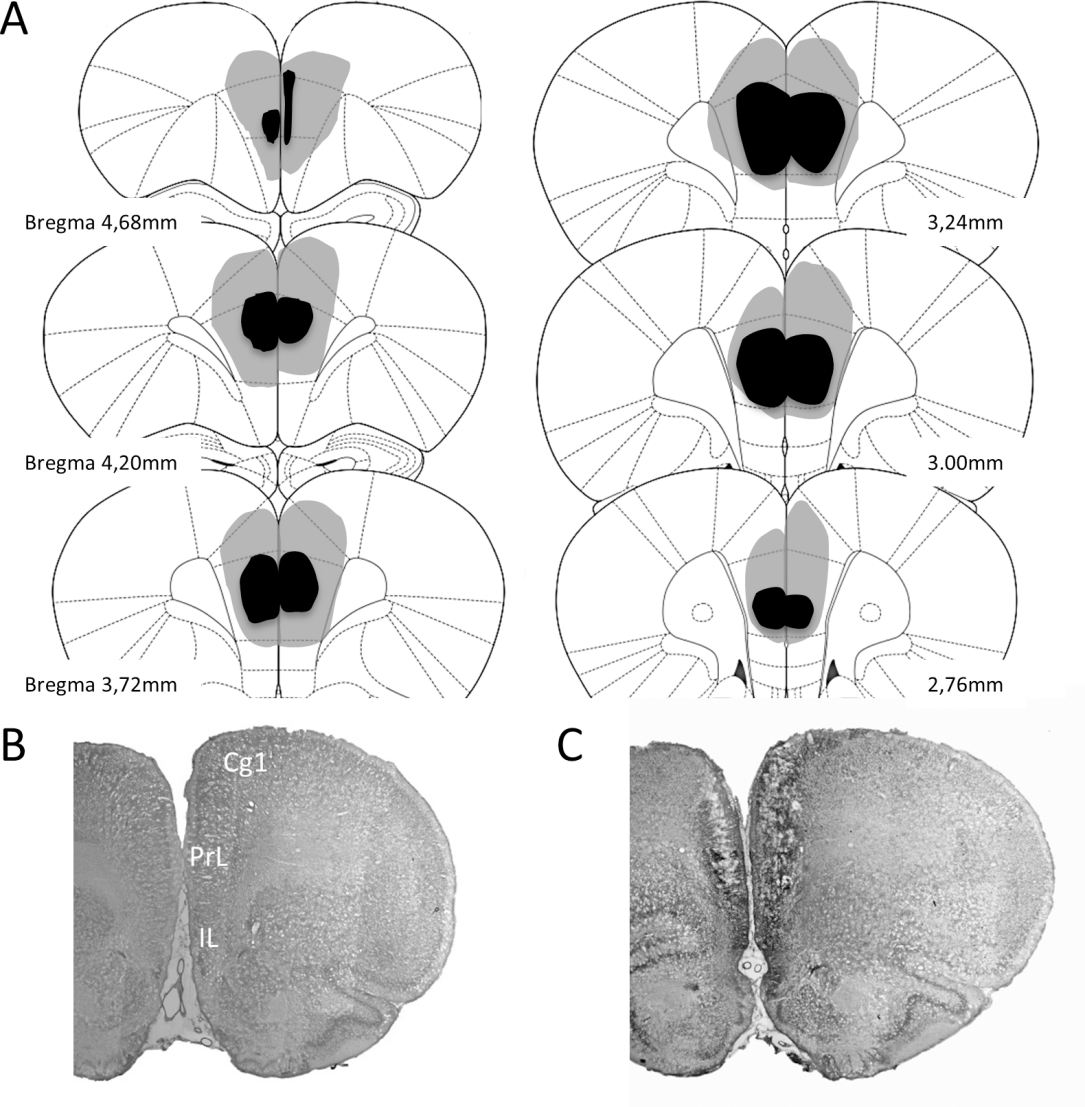
**A.** Reconstruction of the mpfc lesions. Similar to dls lesion, the largest and smallest lesion are shown in pale and dark shading respectively. **B** and **C**. show photomicrograph of a no lesioned coronal brain section and another one with a excitotoxic lesion of mpfc. Cg1: cingulate cortex, area 1; IL: infralimbic cortex; PrL: prelimbic cortex.

Upon completion of behavioral testing, rats were deeply anesthetized and perfused transcardially with a fixative solution (10% formalin in phosphate buffer 0.1 M, pH 7.4). The brains were then removed from the skull and placed in 10% formalin and buffered for 3–4 days. Next, the brains were cut with a microtome at the coronal plane at 50 μm thickness oriented according to the atlas of Paxinos & Watson [32] and Nissl stained for histological analysis. Specifically, we stained the tissue with cresyl violet method. This allows to determine the extent of dls and mpfc lesion in the experimental groups. Lesions were quantified in dls and mpfc groups. Animals included in this study showed lesions between 52–94% of damage in dls (Fig 1) and 56–89% in mpfc (Fig 2), and without significant damage to the adjacent structures.

### Data analysis

Preexposure phase: repeated measure analysis of variance (ANOVA) test was performed to assess the effects of the trials and their interaction with lesion and exposure (between subject factors). The factors for the analyses were lesion, exposure and trials of saccharin consumption. Conditioning and test phase included the factors lesion and exposure. Significant interactions were analyzed with simple main effects based on the pooled error term (Bonferroni corrections). In addition, simple effects in test phase were analyzed with Student’s t test. Statistical significance was set at p<0.05.

## Results

### Experiment 1A. Extended exposure to future CS in animals with dls lesion

#### Preexposure phase

**Fig 3:**
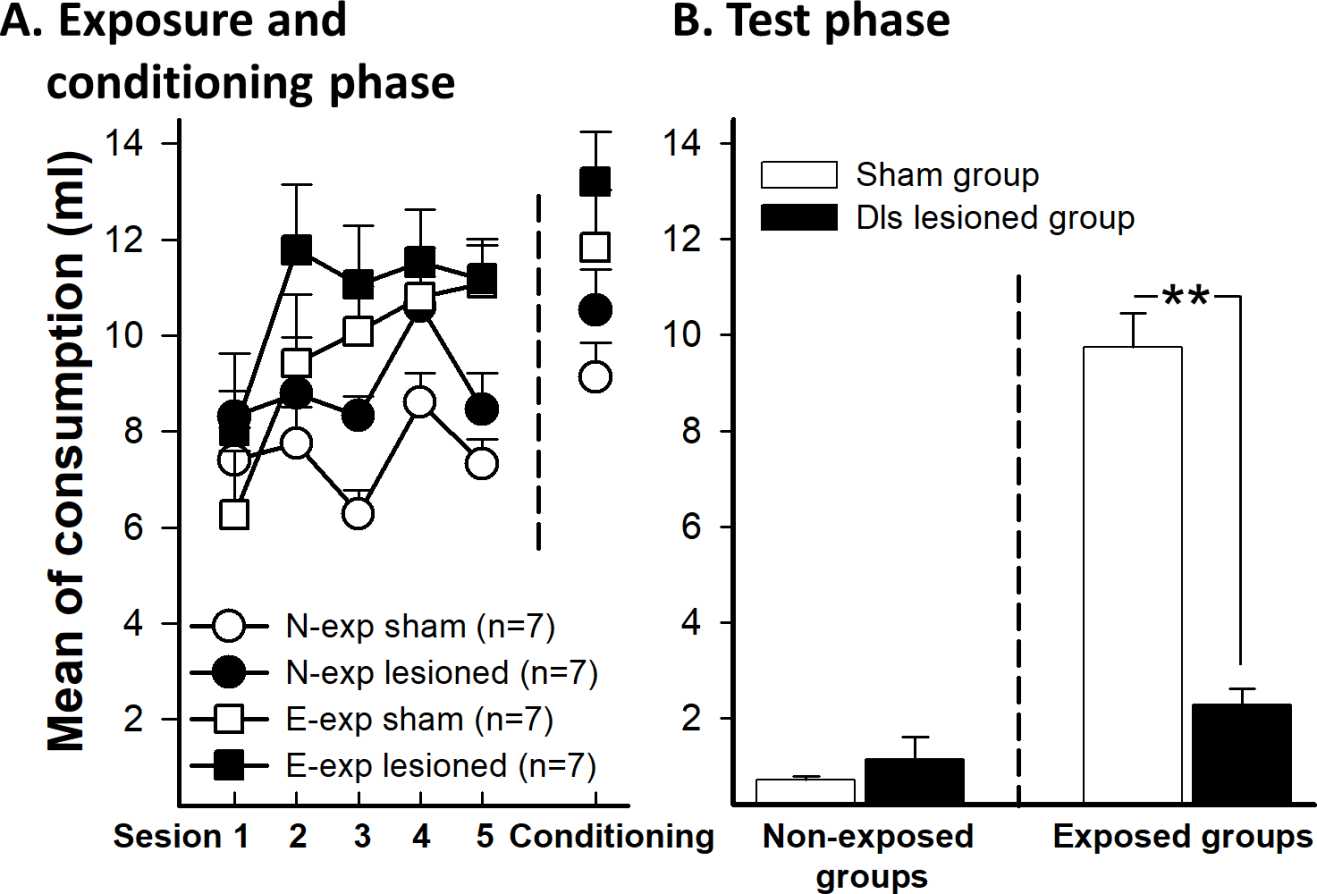
Saccharin intake for sham and dls lesioned animals in E-exp and N-exp groups. E-exp groups was exposed to saccharin for five days before conditioning day. N-exp were only exposed to saccharin the conditioning day. **A** (left). The graphic shows the five sessions of preexposure phase for the 10.00 am session and (right) saccharin intake during conditioning phase. **B**. Effects of dls lesion on mean saccharin intake in extended exposure groups (E-exp) to the future CS during the test phase. Error bars represent SEMs. Asterisks indicate p<0.01. N-exp: non-exposed groups, E-exp: extended exposure groups.

The mean liquid consumption across preexposure trials as a function of the preexposure and the lesion conditions showed differences between groups. A mixed 5 (trials) x 2 (preexposure) x 2 (lesion) ANOVA revealed a significant main effect of trials, F(4,96) = 8.89, p<0.01, and a main effect of preexposure, F(1,24) = 11.73, p <0.01. These data indicate that animals increased the consumption across the sessions, probably because of the effect of neophobia was reduced across sessions. However, the main effect of lesion was not significant on comsumption, F(1, 24) = 4.05, p = 0.055 (Fig 3A). The Trials x Preexposure interaction was significant, F(4,96) = 5.84, p<0.01, ƞ_p_^2^ = 0.196, probably reflecting a general habituation of neophobia effect in the E-exp group. No other interaction was significant (all ps>0.05; Fig 3A).

#### Conditioning phase

The analysis of the conditioning session with an ANOVA 2 x 2 (preexposure x lesion) revealed a significant effect of the preexposure on consumption, F(1,24) = 9.39, p<0.01, since animals of both E-exp groups consumed more solution that N-exp groups. However, neither the effect of the lesion F(1, 24) = 2.79, p = 0.10 nor the interaction Preexposure x Lesion, F(1, 24) = 0.47, p = 0.83, were significant (Fig 3A).

#### Test phase

Fig 3B shows mean liquid intake in test trials as a function of preexposure and lesion. To analyze the consumption during the test, a 2 x 2 ANOVA (preexposure x lesion) was conducted. The analysis revealed a significant main effect of preexposure, F(1, 24) = 68.09, p<0.01, and lesion, F(1, 24) = 19.91, p<0.01. The interaction Preexposure x Lesion was also significant, F(1,24) = 28.73, p<0.01, ƞ_p_^2^ = 0.517. The analysis of the interaction revealed that there were differences between E-exp and N-exp groups in the Sham condition t(12) = 12.48, p<0.01. However, LI effect was not observed in the lesion condition, t(12) = 1.88, p = 0.085. The E-exp sham group drank more saccharin than the lesion group, t(12) = 5.10, p<0.01. However, the N-exp groups did not differ between themselves, t(12) = 0.85, p = 0.40.

### Experiment 1B. Dls lesion and limited exposure to future CS

#### Preexposure phase

**Fig 4:**
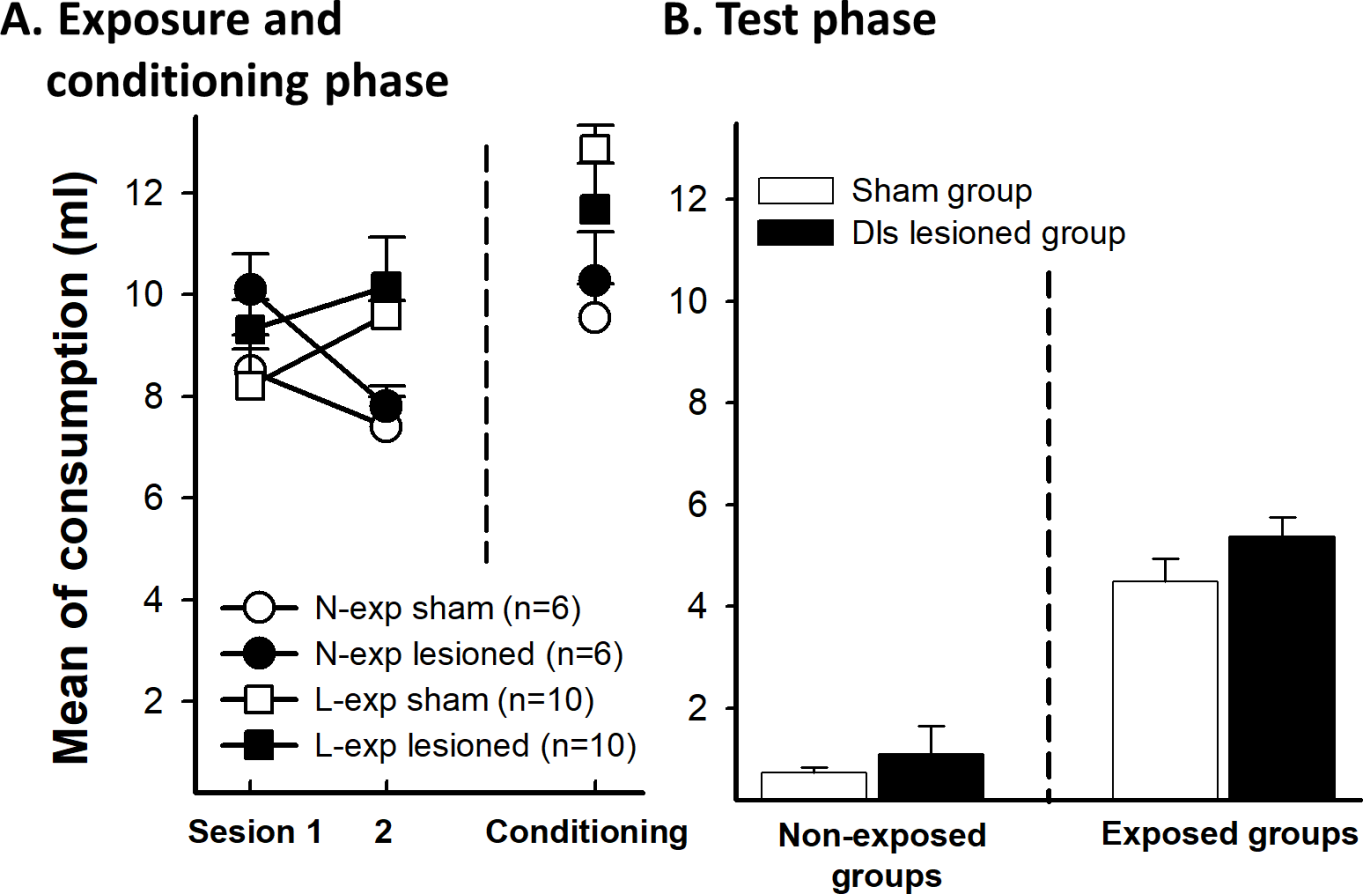
A. (left) Mean saccharin intake (ml) during the two sessions of preexposure and (right) conditioning phase for sham and dls lesioned animals (L-exp and N-exp). The graph shows the consumption for preexposed and non-preexposed groups to the future CS. L-exp groups was exposed to saccharine two days before conditioning and N-exp groups were similar to experiment 1A. **B.** Test phase. Effects of dls lesion on mean saccharin intake in limited exposure of the future CS (L-exp). Error bars represent SEMs. N-exp: non exposed groups, L-exp: limited exposure groups.

An ANOVA 2(trials) x 2(preexposure) x 2(lesion) was conducted on the mean of liquid consumed across the preexposure trials. Similar to Experiment 1, this analysis showed an interaction effect of Trials x Preexposure, F(1, 28) = 12.38, p<0.01, ƞ_p_^2^ = 0.267, due to the preexposure groups drinking more liquid than the non-exposure groups in the second trial, F(1, 28) = 10.49, p<0.01 (Fig 4A). Rats of L-exp condition increased consumption of saccharin from trial 1 to trial 2, F(1, 28) = 4.53, p = 0.042. In contrast, the water intake decreased in the N-exp condition the second day of exposure, F(1, 28) = 7.84, p<0.01; however, consumption of both groups remained high. No other effect was significant (all ps>0.05; Fig 4A).

#### Conditioning phase

Consumption on the conditioning day was analyzed by an ANOVA 2 (preexposure) x 2 (lesion). We found the same effect as for Experiment 1. There was a main effect of preexposure, F(1, 28) = 8.62, p<0.01, because saccharin consumption by the N-exp groups was lower than the consumption of the L-exp groups (Fig 4A). No other effect approached significance (all ps>0.05).

#### Test phase

Fig 4B shows the results of the consumption test. LI effect was evident for sham and lesioned animals. An ANOVA 2 (preexposure) x 2 (lesion) conducted on the mean amount of saccharin consumed in the test trials revealed a significant main effect of preexposure, F(1, 28) = 82.31, p<0.01, reflecting a general LI effect. Neither the main effect of lesion (p = 0.16) nor the interaction Preexposure x Lesion (p = 0.56) were significant, showing that the lesion did not affect the preexposure effect.

### Results experiment 2A. Extended exposure to future CS after mpfc lesion

#### Preexposure and conditioning phase

**Fig 5:**
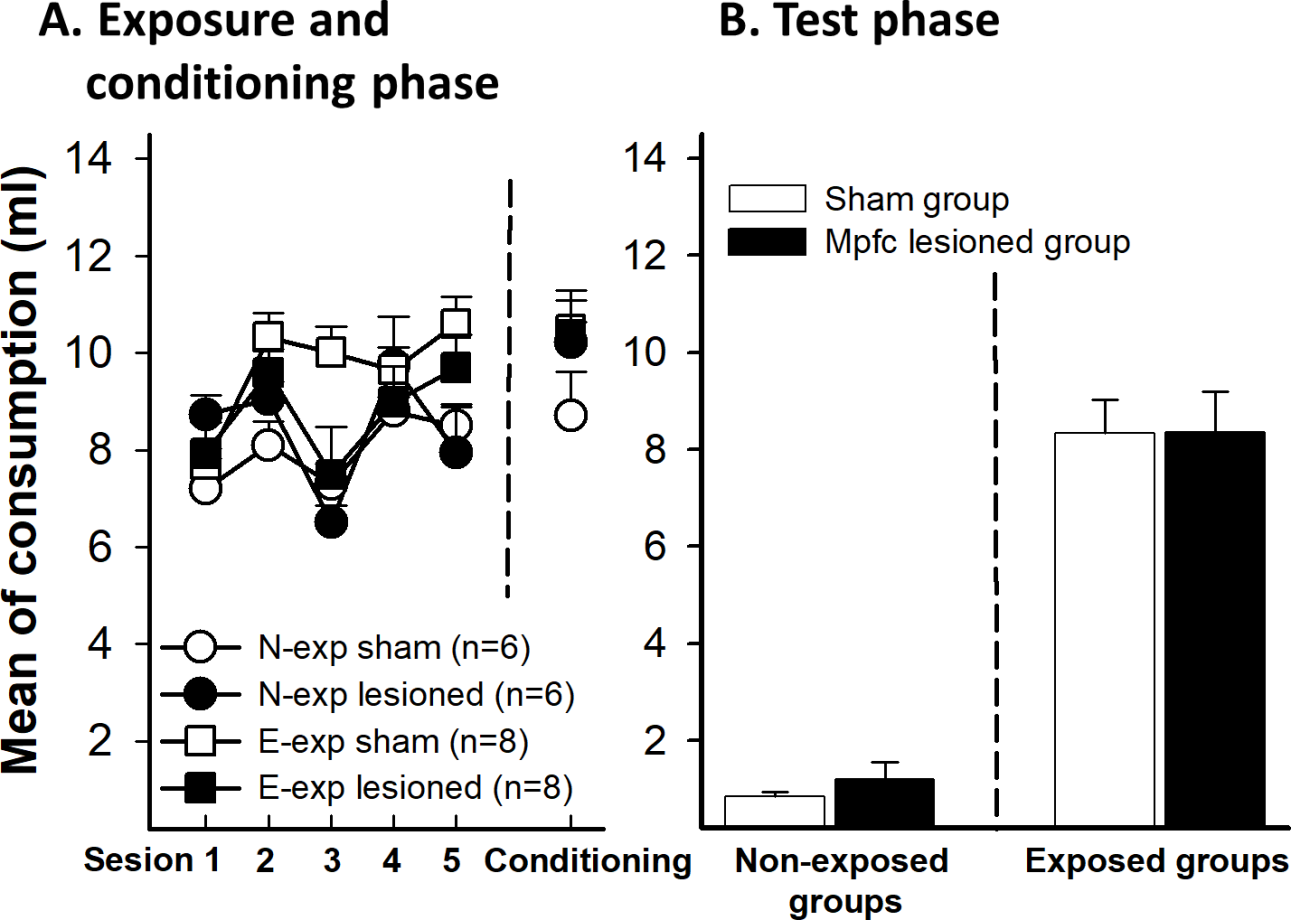
Saccharin intake for sham and mpfc lesioned animals (E-exp and N-exp). As experiment 1, **A** (left) shows the five sessions of preexposure phase and (right) saccharin intake during conditioning phase. **B**. Effects of mpfc lesion in extended exposure groups (E-exp) during the test phase. Error bars represent SEMs. N-exp: non exposed groups, E-exp: extended exposure groups.

A repeated measured ANOVA 5x2x2 using preexposure trials as a within-subjects factor and preexposure and lesion as between-subjects factors showed a significant main effect of preexposure, (F(1, 25) = 6.715, p = 0.016, of trials, F(4,100) = 8.60, p<0.01, an interaction between these factors, F (4, 100) = 3.702, p<0.01, ƞ_p_^2^ = 0.129, and a trial x lesion interaction, F(4, 100) = 3.58, p<0.01, ƞ_p_^2^ = 0.125. Simple main effect analysis revealed that the extended exposure groups consumed more liquid than the non-exposure groups in trials 2, 3, and 5 (all ps<0.05) probably due to the preference of animals for sweet taste of saccharin. The lesioned groups drank less saccharin than the sham groups in trial 3 (p<0.01) but there were no differences in any other trial (Fig 5A).

On the conditioning day, an ANOVA 2x2 conducted using preexposure and lesion as factors found no significant effect of preexposure (F(1,25) = 1.006, p = 0.326), no effect of lesion F(1, 25) = 0.476, p = 0.497, nor a preexposure x lesion interaction, F(1, 25) = 0.744, p = 0.397 (Fig 5A).

#### Test phase

Fig 5B shows the mean of saccharin consumption in the test trial as a function of the preexposure and the lesion conditions. There was a LI effect in both lesioned and sham groups. An ANOVA 2x2 with preexposure and lesion as factors showed main effect of Preexposure, F(1, 25) = 138.01, p<0.01. None other effect was significant (lesion, p = 0.752 and preexposure x lesion, p = 0.658) indicating that the LI effect was not affected by the mpfc lesion.

### Results experiment 2B: Limited exposure to future CS after mpfc lesion

#### Preexposure and conditioning phase

**Fig 6:**
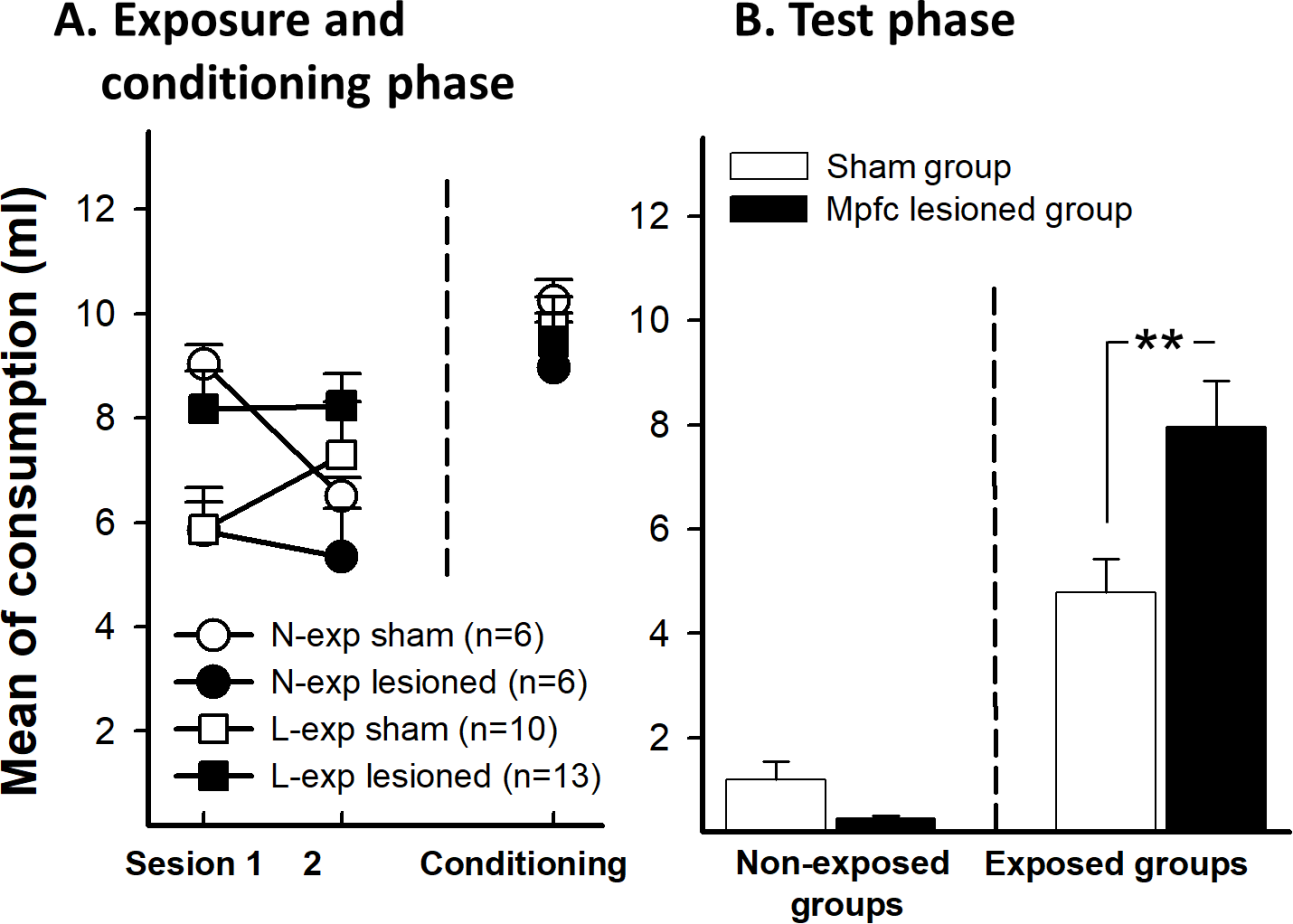
**A.** As experiment 2, the graph shows the mean saccharin intake during the two sessions of preexposure (left) and conditioning phase (right) for sham and mpfc lesioned animals (L-exp and N-exp). **B.** Mean of saccharin intake in limited exposure (L-exp) of the future CS in sham and mpfc lesioned groups. Error bars represent SEMs. Asterisks indicates p<0.01. N-exp: non exposed groups, L-exp: limited exposure groups.

Fig 6A shows mean liquid consumption across preexposure trials as a function of the preexposure and the lesion conditions. A mixed 2 (trials) x 2 (preexposure) x 2 (lesion) ANOVA with trials as the within-subject factor revealed a significant preexposure x lesion interaction effect, F(1,31) = 5.99, p = 0.02, ƞ_p_^2^ = 0.145. This effect was due to the limited exposure mpfc group drank more liquid than non-exposure group (p = 0.019). The interaction trials x preexposure was also significant, F(1,31) = 4.38, p = 0.04, ƞ_p_^2^ = 0.150, reflecting a habituation of the neophobia to the new flavor. No other effect was significant (all ps>0.05). During conditioning phase, there were not any significant effect (all ps>0.05, see Fig 6A).

#### Test phase

Fig 6B displays the results of the test. Lesioned animals showed a higher LI effect than sham animals. To analyze the consumption in the test, an ANOVA 2 (preexposure) x 2 (lesion) was conducted. The analysis revealed a significant main effect of preexposure, F(1, 31) = 48.61, p<0.01, reflecting a general LI effect. The main effect of lesion was not significant (p>0.05). The interaction Preexposure x Lesion was significant, F(1,31) = 7.15, p = 0.012, ƞ_p_^2^ = 0.188. The analysis of this two-way interaction revealed that mpfc lesion exclusively affected the preexposure learning. There were differences between L-exp and N-exp groups in sham F(1, 31) = 8.83, p<0.01 and lesioned conditions, F(1, 31) = 48.74, p<0.01. In regard to the L-exp, lesioned group drank more saccharin than the sham group, F(1, 31) = 13.84, p<0.01. However, the N-exp groups did not differ between them, F(1, 31) = 0.36, p = 0.55.

## Discussion

The results from the present experiments show a selective role for the mpfc and dls in LI. Lesions to dls structure did not disrupt LI after only a few trials of preexposure to saccharin (Experiment 1B). However, an effect of the dls lesion was revealed by the absence of LI in the lesioned group that received many exposures to saccharin prior to the conditioning trial (Experiment 1A). In contrast, mpfc animals showed an increased LI with a limited exposure to future CS (Experiment 2B) and a normal LI with the extended training (Experiment 2A). Likewise, we observed normal conditioning in lesioned animals without exposure to the future CS, which indicates that neither the mpfc nor dls are involved in the formation of the CS-US association in the CTA paradigm. These results demonstrate that mpfc and dorsal striatum could have a functional connectivity necessary for LI expression; probably based on the control of attentional processes. Previous work has shown the dorsal striatum and mpfc are involved in LI in different paradigms [23, 33, 34], but this study identifies a specific region of dorsal striatum and mpfc in the control of LI expression with different exposure to the future CS.

Several studies have investingated he involvement of mpfc in LI. For instance, Lacroix et al. [35] trained animals in a lick suppression paradigm to test the effects of dopamine agonist and antagonist administration in mpfc; and they did not find any effect on LI. Our results are congruent with this study, since we also found that LI is not disrupted by damage to the mpfc. Our results are also congruent with a previous study by George et al. [23] testing different areas of mpfc with a conditioned emotional response paradigm. Even though it is difficult to draw clear conclusions when comparing results from conditioned emotional response and taste aversion paradigms, it is noteworthy that this study found a clear LI following mpfc lesions, and an increased LI after lesion of ventromedial prefrontal cortex. In contrast, Nelson et al. [36] found a LI release after dopaminergic depletion in prelimbic cortex with the same paradigm. These results are similar to present data, since the exposure to future CS could be similar to a limited exposure group in our study. Based on Weiner’s model [37], they maintained that prefrontal cortex was concerned with a switching mechanism between the responses associated with an ambiguous stimulus. If this is so, our results suggest that this mechanism will only act in those situations where the preexposure learning is controlled. However, when the preexposure learning is under control of automatic processes, the prefrontal switching mechanisms will not be activated. In this regard (according to this suggestion), Killcross & Coutureau [38] found that animals with lesions of the prelimbic area lost the sensitivity to changes in the value of reward after both limited or extended training. These results could point to prefrontal cortex involvement in coordinating controlled and automatic response, and the prelimbic and infralimbic region should play an important role in the active inhibition of mechanisms that produce habitual response. Thus, it is plausible to explain the effects on LI as possible fluctuations in attentional processes. This idea emerges from the present results, where attentional processes change with the training sessions, and they may be modulated by different neural structures.

The results from these experiments are in line with those reported by Díaz et al. [10], who blocked activity in the dls only during the test stage of the experiment, and found a disruption of LI with many, but not a few preexposure trials. In light of these findings, it appears that the effect described is reliable, and can also be found when the dls is made inactive by being lesioned before starting the experiment. Moreover, by disrupting activity in the dls before the preexposure rather than immediately before the test trial (as was the case for Díaz et al. [10]), it is not possible to attribute the disruption of LI in the present study to a possible change in the internal context or physiological state before the test trial.

Thus the present results point to the conclusion that two distinct neural mechanisms are responsible for LI in conditioned taste aversion. One mechanism might require an intact dls, and results in a loss of attention to cues with extended exposure. The second mechanism would be more complex and would involve the mpfc, resulting in a LI after a limited amount of exposure to a cue. Presumably with extended exposure the influence of the first mechanism (or the mechanism involved in automatic processes) in animals without lesions suppresses the influence of the second process (or controlled), and thus results in LI with both limited and extended exposure to saccharin prior to taste aversion conditioning.

Dorsal striatum and mpfc play an important role in the cognitive control of actions [18, 39–41], and numerous neural mechanisms are involved in the underlying attentional processes. In this regard, Díaz et al. [10] presented some findings that support this hypothesis in animals with dms lesion. They found that lesion of this structure had effects on a possible suppression of the influence of the automatic processes. Furthermore, they found the expression of LI after extended exposure was similar to that after a limited exposure to a cue, suggesting a release of the controlled processes after dms lesion. These data are also congruent with Molero-Chamizo results [42]. This study found no effect in LI after dorsal striatum lesion. We found the same result with a limited expose to future CS, but not with an extended exposure. Taken as a whole, dorsal striatum could be involved in habituation to future CS, with dms and dls playing different roles in LI. While dms is involved in inhibition of the second mechanism (controlled processes), dls would be essential to the expression of the automatic one. However, the region that is responsible for this second mechanism remained to be identified. Results from our experiments identify the mpfc as the region responsible for this mechanism. It is probable that each mechanism involves a distributed network of corticostriatal circuits [18, 19, 31, 43–47], emphasizing specific circuits. It is also possible that attentional processes are closely linked to other cortical and subcortical phenomenon [23, 48] where the dorsal striatum and mpfc play an important role in the activity of both mechanisms.

In summary, our results provide the first demonstration of a specific corticostriatal activity in LI. These data expand our understanding of how the mpfc and dls could work to inhibit each other in regard to control the attentional processes. Given the importance of LI to gate out sensory and cognitive information [49–51], these results have important implications in understanding possible risk factors and symptoms of severe mental illness.

## Supporting information

S1 Table Saccharine intake along sessions for the four experiments. (PDF)
