# Supplementary material for: Different involvement of medial prefrontal cortex and dorso-lateral striatum in automatic and controlled processing of a future conditioned stimulus
Source: PLoS One. 2017 Dec 14;12(12):e0189630. doi: 10.1371/journal.pone.0189630 (PMC5730208; doi:10.1371/journal.pone.0189630)
Supplement: S1 Table — (PDF) [file pone.0189630.s001.pdf]

Experiment 1

	day 1	day 2	day 3	day 4	day 5	conditioning	test
N-exp sham	–	–	–	–	–	11,57	0,7
N-exp sham	–	–	–	–	–	10,7	0,9
N-exp sham	–	–	–	–	–	6,6	0,7
N-exp sham	–	–	–	–	–	10,6	0,4
N-exp sham	–	–	–	–	–	8,6	0,5
N-exp sham	–	–	–	–	–	7,16	1,1
N-exp sham	–	–	–	–	–	8,71	0,8
N-exp dls lesion	–	–	–	–	–	9,17	0,86
N-exp dls lesion	–	–	–	–	–	9,15	3,88
N-exp dls lesion	–	–	–	–	–	14,71	0,45
N-exp dls lesion	–	–	–	–	–	12,04	1,38
N-exp dls lesion	–	–	–	–	–	7,92	0,56
N-exp dls lesion	–	–	–	–	–	9,98	0,42
N-exp dls lesion	–	–	–	–	–	10,72	0,44
L-exp sham	–	–	–	10,97	10,43	13,93	4,1
L-exp sham	–	–	–	11,36	9,42	11,95	5,73
L-exp sham	–	–	–	6,01	9,24	14,19	4,32
L-exp sham	–	–	–	8,98	10,24	11,5	5,16
L-exp sham	–	–	–	8,36	11,27	15,01	2,77
L-exp sham	–	–	–	9,62	9,68	13,76	7,11
L-exp sham	–	–	–	9,34	9,14	12,36	3,39
L-exp sham	–	–	–	5,75	8,1	11,92	5,9
L-exp sham	–	–	–	4,22	9,92	10,23	3,24
L-exp sham	–	–	–	7,42	8,58	13,8	3,22
L-exp dls lesion	–	–	–	7,54	16,36	17,34	4,42
L-exp dls lesion	–	–	–	9,13	9,81	9,3	3,35
L-exp dls lesion	–	–	–	10,92	10,67	10,94	4,7
L-exp dls lesion	–	–	–	13,33	14,37	15,91	5,37
L-exp dls lesion	–	–	–	9,89	9,37	10,54	6,23
L-exp dls lesion	–	–	–	7,04	8,73	9,49	7,9
L-exp dls lesion	–	–	–	10,03	9,77	9,65	5,93
L-exp dls lesion	–	–	–	9,25	8,03	13,25	5,13
L-exp dls lesion	–	–	–	8,34	8,69	9,92	5,7
L-exp dls lesion	–	–	–	7,7	5,89	10,46	5,08
E-exp sham	4,2	9,9	9,2	9,5	12,8	8,1	9,2
E-exp sham	5,1	10,1	9,4	12,6	11,4	12,8	12,4
E-exp sham	0,6	12,3	14,7	15,3	14,3	15,7	11,3
E-exp sham	8,3	1,5	10,2	7,8	11,6	10,7	6,9
E-exp sham	11,7	12,6	13,8	11,7	14,1	16,3	10,7
E-exp sham	8,27	8,19	7,86	9,55	8,8	10,07	9,61
E-exp sham	5,7	11,4	9,97	10,38	9,61	9,27	8,08
E-exp dls lesion	9,3	10,5	9,3	10,7	9,9	12,2	3,16
E-exp dls lesion	6,3	8,8	10,2	8,7	9	9	2,18

E-exp dls lesion	6	8	7,9	8,7	10,7	12,4	3,2
E-exp dls lesion	7,2	12,2	13	17,3	13,8	16,4	9,3
E-exp dls lesion	11,4	14,5	14,7	12,1	10,8	14,5	2,4
E-exp dls lesion	8,2	16,7	15	15,2	14,9	15	1,1
E-exp dls lesion	6,9	12,2	9,3	13,8	11,8	16,1	1,7

Experiment 2

	day 1	day 2	day 3	day 4	day 5	conditioning	test
N-exp sham	–	–	–	–	–	11,72	1,52
N-exp sham	–	–	–	–	–	9,84	0,57
N-exp sham	–	–	–	–	–	9,66	0,87
N-exp sham	–	–	–	–	–	11,39	2,81
N-exp sham	–	–	–	–	–	9,25	0,88
N-exp sham	–	–	–	–	–	9,69	0,63
N-exp mpc lesion	–	–	–	–	–	10,23	0,61
N-exp mpc lesion	–	–	–	–	–	12,45	0,52
N-exp mpc lesion	–	–	–	–	–	7,07	0,2
N-exp mpc lesion	–	–	–	–	–	8,95	0,49
N-exp mpc lesion	–	–	–	–	–	8,5	0,51
N-exp mpc lesion	–	–	–	–	–	6,56	0,46
L-exp sham	–	–	–	3,82	6,74	9,24	3,59
L-exp sham	–	–	–	6,19	7,12	8,3	2,35
L-exp sham	–	–	–	4,9	1,81	9,86	2,32
L-exp sham	–	–	–	5,99	1,56	6,88	7,25
L-exp sham	–	–	–	6,66	5	9,09	5,64
L-exp sham	–	–	–	4,22	9,92	10,23	3,24
L-exp sham	–	–	–	11,78	13,08	9,8	7,9
L-exp sham	–	–	–	4,05	8,86	12,78	3,52
L-exp sham	–	–	–	7,42	8,58	13,8	3,22
L-exp sham	–	–	–	7,99	9,41	8,71	6,31
L-exp mpc lesion	–	–	–	6,65	8,16	8,41	7,36
L-exp mpc lesion	–	–	–	9,47	5,87	9,6	8,76
L-exp mpc lesion	–	–	–	7,28	6,98	8,89	5,47
L-exp mpc lesion	–	–	–	2,62	6,96	8,91	6
L-exp mpc lesion	–	–	–	7,88	9,81	11,49	6,79
L-exp mpc lesion	–	–	–	7,34	9,53	11,2	4,23
L-exp mpc lesion	–	–	–	8,18	6,22	7,8	5,54
L-exp mpc lesion	–	–	–	8,19	6,31	10,98	9,05
L-exp mpc lesion	–	–	–	7,7	9,26	9,9	7,65
L-exp mpc lesion	–	–	–	7,6	7,6	10,18	10,9
L-exp mpc lesion	–	–	–	14,08	7,84	6,11	6,4
L-exp mpc lesion	–	–	–	8,87	9,26	6,6	9,31
L-exp mpc lesion	–	–	–	8,96	13,32	12,04	15,51

E-exp sham	8	10	11,33	7,3	10,74	9,69	11,05
E-exp sham	8	8,6	9,7	8,29	9,45	7,01	9
E-exp sham	7,08	11,6	12,46	10,38	12,3	15,06	5,66
E-exp sham	8,27	8,19	7,86	9,55	8,8	10,07	9,61
E-exp sham	5,7	11,4	9,97	10,38	9,61	9,27	8,08
E-exp sham	8,97	10,52	9,75	10,54	9,82	10,75	9,41
E-exp sham	8,34	10,14	8,22	9,52	10,53	10,37	9,27
E-exp sham	7,18	12,2	10,79	11,37	13,59	11,66	5,38
E-exp mpc lesion	5,22	9,58	3,95	5,95	7,43	9,03	5,22
E-exp mpc lesion	5,77	11,3	5,78	12,67	12,15	14,17	5,77
E-exp mpc lesion	7,44	9,56	8,64	9,3	11,37	10,37	7,44
E-exp mpc lesion	8,07	10,97	4,95	7,06	6,99	11,47	8,07
E-exp mpc lesion	9,7	6,79	5,46	7,82	8,01	8,75	9,7
E-exp mpc lesion	8,7	9,97	9,5	10,23	10,35	11,52	8,7
E-exp mpc lesion	10,6	10	11,33	9,26	11,2	10,19	12,84
E-exp mpc lesion	8	8,6	10,37	9,69	10,09	7,38	9,07
